# BET inhibitors potentiate melanoma ferroptosis and immunotherapy through AKR1C2 inhibition

**DOI:** 10.1186/s40779-023-00497-1

**Published:** 2023-12-04

**Authors:** Yu Meng, Hui-Yan Sun, Yi He, Qian Zhou, Yi-Huang Liu, Hui Su, Ming-Zhu Yin, Fu-Rong Zeng, Xiang Chen, Guang-Tong Deng

**Affiliations:** 1 Department of Dermatology, Xiangya Hospital, Central South University, Changsha, 410008 China; 2 National Engineering Research Center of Personalized Diagnostic and Therapeutic Technology, Changsha, 410008 China; 3 Furong Laboratory, Changsha, 410008 China; 4 Hunan Key Laboratory of Skin Cancer and Psoriasis, Hunan Engineering Research Center of Skin Health and Disease, Xiangya Hospital, Central South University, Changsha, 410008 China; 5 National Clinical Research Center for Geriatric Disorders, Xiangya Hospital, Changsha, 410008 China; 6 Department of Breast Reconstruction, Tianjin Medical University Cancer Institute and Hospital, Tianjin, 300202 China; 7 Department of Oncology, Xiangya Hospital, Central South University, Changsha, 410008 China

**Keywords:** Melanoma, Bromodomain and extra terminal domain (BET) inhibitor, Ferroptosis, Cell death, AKR1C2, Immunotherapy

Dear Editor,

Ferroptosis, an iron-dependent form of cell death driven by overwhelming lipid peroxidation, represents a vulnerability in cancers, and therapeutic strategies to further potentiate ferroptosis hold great potential for melanoma treatment.

To systematically identify drugs that sensitize ferroptosis, we initially calculated ferroptosis score (FPS) using our published algorithm model [1], and conducted spearman correlation analysis between FPS and the cell sensitivities to various anti-cancer drugs across 859 cancer cell lines. Remarkably, drugs targeting epigenetic regulators were significantly associated with high FPS, especially bromodomain and extra-terminal domain (BET) inhibitors (I-BET151, JQ1, and GSK525762A) (Fig. 1a). Further investigations revealed a strong synergy in melanoma cells when BET inhibitors (JQ1, NHWD-870, OTX015, and I-BET151) were combined with the ferroptosis inducer RSL3, an inhibitor of glutathione peroxidase 4 (GPX4), with combination index values less than 1 and fewer colony numbers (Additional file 1: Fig. S1a-j). Consistently, BET inhibitors also sensitize melanoma cells to genetic inhibition of *GPX4* (Additional file 1: Fig. S1k-s). Notably, the cytotoxicity of the co-treatment of BET inhibitors and RSL3 could be completely abrogated by the ferroptosis inhibitor ferrostatin-1 and the iron chelator deferoxamine, but not by inhibitors of apoptosis (Z-VAD-FMK), necroptosis [necrostatin-1s (Nec-1s)], or autophagy [chloroquine (CQ)] in melanoma cells (Fig. 1b, Additional file 1: Fig. S2a). The combined treatment triggered prominent ferroptosis-related characteristics, including more lipid peroxidation (Fig. 1c, Additional file 1: Fig. S2b-c), and shrunken mitochondria with increased membrane density (Fig. 1d). Moreover, the BET inhibitors-enhanced cell death in *GPX4*-deficient melanoma cells was restored by ferroptosis inhibitors (Additional file 1: Fig. S2d-g). These results suggest that BET inhibitors potentiate *GPX4* inhibition-induced ferroptosis in melanoma.

**Fig. 1:**
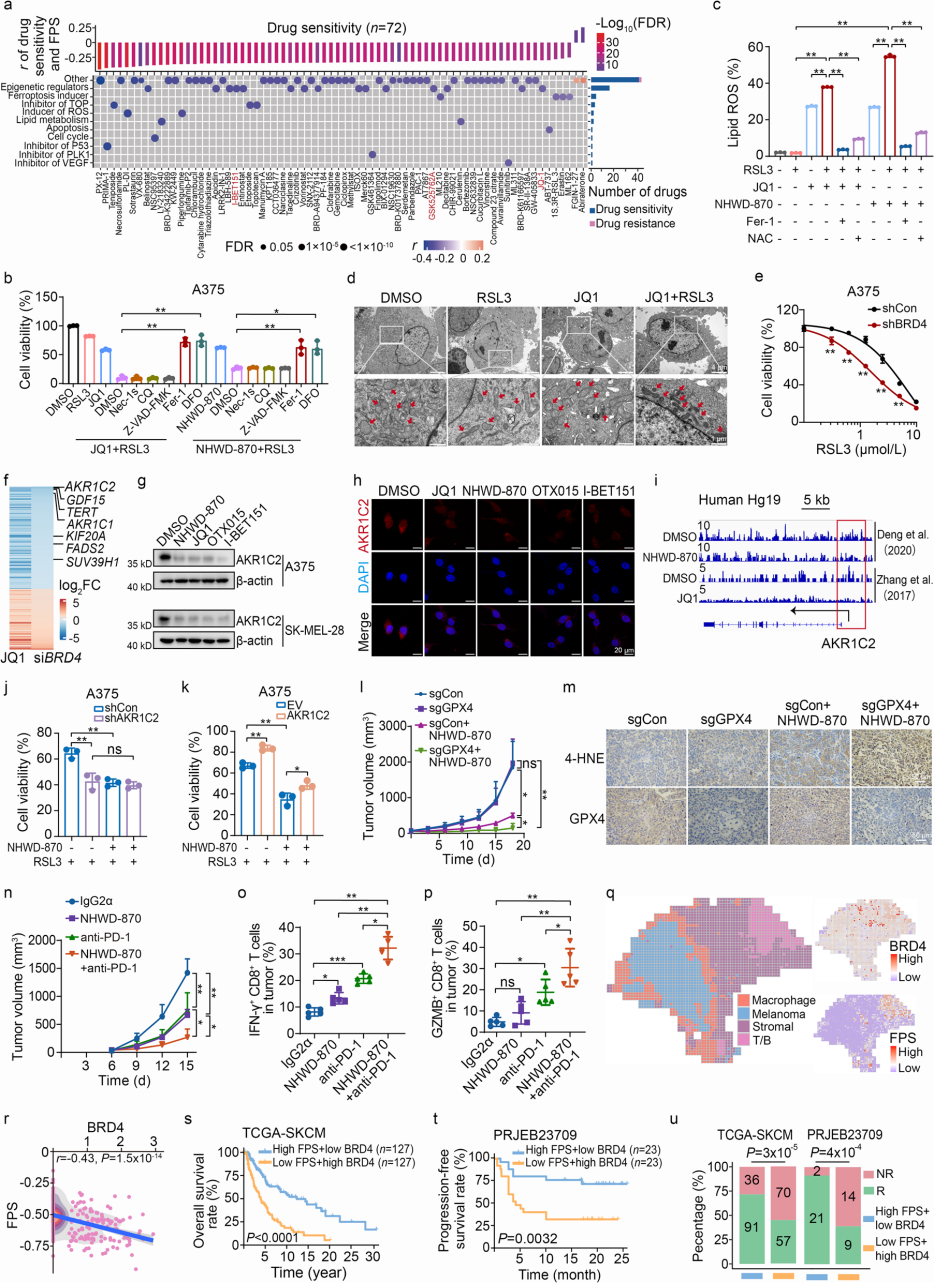
BET inhibitor-mediated downregulation of AKR1C2 sensitizes melanoma to ferroptosis induced by GPX4 inhibition. **a** Signaling pathways targeted by drugs that are sensitive (blue) or resistant (red) to the ferroptosis score (FPS) from CTRP database. Drug names are listed on the *x*-axis and the signaling pathway targeted by the drug on the *y*-axis. The upper bar plot denotes the Spearman correlation between FPS and drug sensitivity. *r* < 0 defines drug sensitivity, *r* > 0 defines drug resistance; the bar plot on the right shows the number of drugs targeting each signaling pathway; The size of the point indicates the significance of the correlation. **b** A375 melanoma cells were pretreated with JQ1 (1 μmol/L) or NHWD-870 (10 nmol/L) for 24 h, and then cotreated with RSL3 (2.5 μmol/L). DMSO, necrostatin-1s (Nec-1s, 10 μmol/L), chloroquine (CQ, 10 μmol/L), Z-VAD-FMK (10 μmol/L), ferrostatin-1 (Fer-1, 4 μmol/L), or deferoxamine (DFO, 100 μmol/L) were added in combination groups for 10 h, and cell viability was assessed. **c** Lipid peroxidation production in A375 cells was measured by flow cytometry using BODIPY-C11. Cells were first treated with 1 μmol/L JQ1 or 10 nmol/L NHWD-870 for 24 h, alone or in combination with 2 μmol/L RSL3, 2 μmol/L RSL3 plus 4 μmol/L Fer-1, 2 μmol/L RSL3 plus 1 mmol/L N-acetyl-cysteine (NAC) for another 6 h as indicated. **d** Transmission electron microscopy of A375 cells pretreated with JQ1 (1 μmol/L) for 24 h and then co-treated with RSL3 (2.5 μmol/L) in combination group for 6 h. Red arrow indicates morphological change of mitochondria. Scale bar = 4 μm (upper), 1 μm (bottom). **e** Dose–response curves of RSL3-induced death in control (shCon) and BRD4 knockdown (shBRD4) A375 cells. **f** Heatmap representation of changes in gene expression in JQ1-treated/siBRD4 versus control A375 cells (*P* < 0.05). Each horizontal line represents one gene, ordered by gene expression. **g** The regulatory effect of BET inhibitors on AKR1C2 expression after 48 h treatment based on Western blotting. DMSO (0.02%); NHWD-870: 10 nmol/L; JQ1: 2 μmol/L; OTX015: 2 μmol/L; I-BET151: 2 μmol/L. **h** Quantification of AKR1C2 expression in A375 cells treated with the same BET inhibitors as **g** by immunofluorescence images. Scale bar = 20 μm. **i** ChIP-seq analysis of BRD4 binding peak in the AKR1C2 promoter in DMSO-treated [2] and NHWD-870-treated melanoma cells (upper) or in DMSO-treated [3] and JQ1-treated cells (lower). Red boxes indicate 2 kb regions around the transcription start site. The arrow indicates the transcription direction. **j** Relative viability of 5 μmol/L RSL3-treated shCon and shAKR1C2 A375 cells at the presence of DMSO or 10 nmol/L NHWD-870. **k** Relative viability of 5 μmol/L RSL3-treated control and AKR1C2 overexpression A375 cells in the presence of DMSO or 10 nmol/L NHWD-870. **l** Tumor growth in control, GPX4 knockout (sgGPX4), NHWD-870, and combination groups. **m** Representative immunohistochemistry images of 4-HNE and GPX4 in the four groups. Scale bar = 50 μm. **n** Tumor growth in the isotype IgG2α, NHWD-870, anti-PD-1 antibody, and combination groups. The percentage of cells expressing IFN-γ (**o**) and GZMB (**p**) in tumor-infiltrating CD8^+^ T cells by flow cytometry analysis. **q** Spatial enhanced-resolution clustering performed by the BayesSpace algorithm at the left panel identified 4 clusters corresponding to the original histopathological annotations. Spatial heatmap shows the level of BRD4 and FPS expression among 4 clusters at the right panel. **r** Scatter plot shows the spearman correlation between the expression level of BRD4 and FPS. Kaplan–Meier curves compare overall survival (TCGA-SKCM) (**s**) and progression-free survival of immune checkpoint inhibitors (ICIs) cohorts (PRJEB23709) (**t**) between the high-FPS + low BRD4 (blue) and low-FPS + high BRD4 (yellow) groups. **u** The proportion of patients with different responses to immunotherapy in the TCGA-SKCM cohort with TIDE-predicted ICB response and the melanoma ICIs cohort (PRJEB23709). R response, including responder, complete response and partial response, NR non-response, including non-responder, stable disease and progressive disease. Quantification data are presented as mean ± SD, and compared using one-way ANOVA in **b-c**, **o-p**, two-way ANOVA in **e**, **j-l**,** n**, log-rank test in **s-t**, and Fisher’s exact test/chi-square test in **u**. AKR1C2 aldo–keto reductase 1C2, BET bromodomain and extra-terminal domain, BRD4 bromodomain-containing protein 4, CQ chloroquine, CTRP Cancer Therapeutics Response Portal, DFO deferoxamine, EV empty vector, FDR false discovery rate, Fer-1 ferrostatin-1, FPS ferroptosis score, GPX4 glutathione peroxidase 4, GZMB granzyme B, 4-HNE 4-hydroxynonenal, ICIs immune checkpoint inhibitors, IFN-γ interferon-γ, NAC N-acetyl-cysteine, Nec-1s necrostatin-1s, NR non-response, PD-1 programmed cell death protein-1, R response, SKCM skin cutaneous melanoma, TCGA the cancer genome atlas, ns non-significant, *P < 0.05, **P < 0.01.

Drug target analysis revealed that *BRD4*, but not *BRD2/3*, was negatively correlated with FPS in melanoma cohorts (Additional file 1: Fig. S3a). Genetic inhibition of *BRD4*, but not *BRD2/3*, enhanced RSL3-induced ferroptosis (Fig. 1e, Additional file 1: Fig. S3b-h), and *BRD4* overexpression resisted ferroptosis in melanoma cells (Additional file 1: Fig. S3i-m), suggesting that BET inhibitors potentiate RSL3-induced ferroptosis by targeting BRD4. RNA-seq analysis demonstrated that *AKR1C2* was most dramatically downregulated by pharmacological and genetic inhibition of *BRD4* among 7 ferroptosis suppressors (Fig. 1f, Additional file 1: Fig. S4a-b), which is consistent with other melanoma datasets after I-BET151 treatment (Additional file 1: Fig. S4c). BET inhibitors could significantly decrease AKR1C2 expression at both mRNA and protein levels (Fig. 1g-h, Additional file 1: Fig. S4d-e). Likewise, genetic inhibition or overexpression of BRD4 suppressed or upregulated the expression of AKR1C2 in melanoma cells, respectively (Additional file 1: Fig. S4f-n). Notably, BET inhibitors could not affect the expression and transcription activity of NRF2, the known transcription factor of AKR1C2 [4], and GPX4 expression (Additional file 1: Fig. S4o-p), ruling out the possibility that BET inhibitors sensitize melanoma ferroptosis through GPX4 or NRF2 repression. ChIP-seq data showed a prominent BRD4 binding peak in the *AKR1C2* gene promoter, while the amplitude of the binding peak was diminished upon NHWD-870 treatment, which is consistent with Zhang et al. [3] ChIP-seq data analysis (Fig. 1i), suggesting that BRD4 transcriptionally regulates *AKR1C2* expression. We previously reported that BET inhibitors suppress STAT3 signaling through the BRD4/IL-6 axis [4]. Inhibiting STAT3 activity by shRNA or the inhibitor stattic significantly suppressed AKR1C2 expression (Additional file 1: Fig. S5a-d). ChIP-seq analysis also demonstrated that STAT3 binds to the *AKR1C2* promoter (Additional file 1: Fig. S5e), suggesting that BET inhibitors directly inhibit *AKR1C2* expression by BRD4, or indirectly by the BRD4/IL-6/STAT3 axis. AKR1C2 was reported to inhibit ferroptosis by degrading lipid peroxides [4], which was validated in our study. Pharmaceutical and genetic inhibition of AKR1C2 sensitized melanoma cells to ferroptosis (Additional file 1: Fig. S6a-h). On the contrary, AKR1C2 overexpression conferred resistance to RSL3-induced ferroptosis in melanoma cells (Additional file 1: Fig. S6i-l). Strikingly, BET inhibitors failed to further potentiate RSL3-induced ferroptosis in the presence of AKR1C2 inhibitors or *AKR1C2* genetic silencing (Fig. 1j, Additional file 1: Fig. S6m-s). Overexpression of *AKR1C2* partially rescued cytotoxicity caused by the co-treatment of BET inhibitors and RSL3 (Fig. 1k, Additional file 1: Fig. S6t-u). These findings suggest that BET inhibitors potentiate RSL3-induced ferroptosis at least partially through AKR1C2 inhibition.

In vivo, *GPX4* knockout exhibited minimal impact on melanoma progression, and NHWD-870 treatment alone moderately inhibited melanoma growth (Fig. 1l, Additional file 1: Fig. S7a-c). However, combining *GPX4* knockout with NHWD-870 significantly impaired melanoma growth and reduced tumor weight (Fig. 1l, Additional file 1: Fig. S7a-d), with markedly increased staining of 4-HNE, an end product of lipid peroxidation (Fig. 1m, Additional file 1: Fig. S7e). Cancer immunotherapy has been regarded as an important ferroptosis-associated pathological model in vivo [6]. We found that BET inhibitor administration potentiated the efficacy of anti-PD-1 antibody (Fig. 1n, Additional file 1: Fig. S7f-i), leading to an elevated proportion of IFN-γ^+^ CD8^+^ and GZMB^+^ CD8^+^ T cells (Fig. 1o-p, Additional file 1: Fig. S7j), despite no increase in total tumor-infiltrating CD8^+^ T cells (Additional file 1: Fig. S7k). These results suggested that BET inhibitors sensitize melanoma to GPX4 inhibition-induced ferroptosis and immunotherapy in vivo. We further observed that *BRD4* expression was negatively associated with FPS in three melanoma single-cell RNA-seq datasets (Additional file 1: Fig. S8a-d), as well as in melanoma regions from spatial transcriptome data (Fig. 1q-r). Notably, *BRD4* is significantly upregulated, while ferroptosis level is downregulated in immunotherapy-resistant malignant melanoma subpopulations (Additional file 1: Fig. S8e). Melanoma patients with high-ferroptosis plus low-*BRD4*/*AKR1C2* predict a better prognosis (Fig. 1s-t, Additional file 1: Fig. S8f) and improved response to immunotherapy (Fig. 1u, Additional file 1: Fig. S8g-h). These findings suggest that BRD4/AKR1C2 is associated with reduced ferroptosis level and poor efficacy of immunotherapy from multi-omics characterization.

Overall, our data illustrate that BET inhibitors potentiate GPX4 inhibition-induced ferroptosis through the dual downregulation of AKR1C2, and provide the rationale for combining BET inhibitors with GPX4 inhibitors or immunotherapy for melanoma treatment (Additional file 1: Fig. S9).

### Supplementary Information

**Additional file 1**: Methods for BET inhibitors potentiate melanoma ferroptosis and immunotherapy through AKR1C2 inhibition. **Fig. S1** BET inhibitors synergize with GPX4 inhibition in melanoma cells. **Fig. S2** Combination of BET inhibitors and GPX4 inhibition causes melanoma ferroptosis. **Fig. S3** BET inhibitors sensitize melanoma cells to RSL3-induced ferroptosis by targeting BRD4. **Fig. S4** BET inhibitors targeted BRD4 regulate AKR1C2 expression by directly targeting its promoter. **Fig. S5** BET inhibitors targeted BRD4 regulates AKR1C2 expression by indirectly targeting the IL-6/STAT3 axis. **Fig. S6** BET inhibitors regulate melanoma susceptibility to RSL3-induced ferroptosis through AKR1C2. **Fig. S7** BET inhibitors potentiate melanoma ferroptosis induced by GPX4 inhibition and immunotherapy in vivo. **Fig. S8** BRD4/AKR1C2 is associated with reduced ferroptosis level and poor efficacy of immunotherapy from multi-omics characterization. **Fig. S9** Schematic depicting BET inhibitor-mediated sensitization to ferroptosis induced by GPX4 inhibition in melanoma cells

## Data Availability

All data generated or analysed during this study are included in this published article and its supplementary information files. Additional data are available upon reasonable request to the corresponding author.

## Abbreviations

AKR1C2: Aldo–keto reductase 1C2
BET: Bromodomain and extra-terminal domain
BRD: Bromodomain-containing protein
CQ: Chloroquine
CTL: Cytotoxic T lymphocyte
CTRP: Cancer therapeutics response portal
CYT: Cytolytic activity
DFO: Deferoxamine
FDR: False discovery rate
Fer-1: Ferrostatin-1
FPS: Ferroptosis score
anti-FRG: Ferroptosis suppressor gene
pro-FRG: Ferroptosis driver gene
GPX4: Glutathione peroxidase 4
GZMB: Granzyme B
4-HNE: 4-Hydroxynonenal
IFN-γ: Interferon-γ
MDSC: Myeloid-derived suppressor cell
MPA: Medroxyprogesterone acetate
MSI: Microsatellite instability
NAC: N-acetyl-cysteine
Nec-1s: Necrostatin-1s
PD-1: Programmed cell death protein-1
RNA-seq: RNA sequencing
SKCM: Skin cutaneous melanoma
STAT3: Signal transducer and activator of transcription 3
TAM: Tumor-associated macrophage
TCGA: The cancer genome atlas
UDCA: Ursodeoxycholate
