# Supplementary material for: BET inhibitors potentiate melanoma ferroptosis and immunotherapy through AKR1C2 inhibition
Source: Mil Med Res. 2023 Dec 4;10:61. doi: 10.1186/s40779-023-00497-1 (PMC10694977; doi:10.1186/s40779-023-00497-1)
Supplement: Supplementary file 1 — Additional file 1: Methods for BET inhibitors potentiate melanoma ferroptosis and immunotherapy through AKR1C2 inhibition. Fig. S1 BET inhibitors synergize with GPX4 inhibition in melanoma cells. Fig. S2 Combination of BET inhibitors and GPX4 inhibition causes melanoma ferroptosis. Fig. S3 BET inhibitors sensitize melanoma cells to RSL3-induced ferroptosis by targeting BRD4. Fig. S4 BET inhibitors targeted BRD4 regulate AKR1C2 expression by directly targeting its promoter. Fig. S5 BET inhibitors targeted BRD4 regulates AKR1C2 expression by indirectly targeting the IL-6/STAT3 axis. Fig. S6 BET inhibitors regulate melanoma susceptibility to RSL3-induced ferroptosis through AKR1C2. Fig. S7 BET inhibitors potentiate melanoma ferroptosis induced by GPX4 inhibition and immunotherapy in vivo. Fig. S8 BRD4/AKR1C2 is associated with reduced ferroptosis level and poor efficacy of immunotherapy from multi-omics characterization. Fig. S9 Schematic depicting BET inhibitor-mediated sensitization to ferroptosis induced by GPX4 inhibition in melanoma cells [file 40779_2023_497_MOESM1_ESM.pdf]

Methods for BET inhibitors potentiate melanoma ferroptosis and immunotherapy through AKR1C2 inhibition

1.1 Cell culture

The human malignant melanoma A375 and SK-MEL-28 cell lines and HEK293T cell lines were cultured in Dulbecco's Modified Eagle’s Medium (Biological Industries, Israel) containing 10% fetal bovine serum (Biological Industries, Israel) and 1% penicillin-streptomycin solution (Beyotime Biotechnology, China) at 37 °C in an incubator with humid air of 5% CO_2. Murine malignant melanoma B16F10 cell lines were cultured in RPMI1640 medium (Biological Industries, Israel). All cell lines were obtained from American Type Culture Collection.

1.2 Chemicals

RSL3 (HY-100218A), Necrostatin 1S (HY-14622A), chloroquine (HY-17589A), Z-VAD-FMK (HY-16658B), ferrostatin-1 (HY-100579), deferoxamine (HY-D0903), liproxstatin-1 (HY-12726), ursodeoxycholate (HY-13771) and medroxyprogesterone acetate (HY-B0469) were purchased from MedChemExpress (USA). JQ1 (S7110), OTX015 (S7360), I-BET151 (S2780), N-acetyl-cysteine (S1623) and static (S7024) were obtained from Selleck China. NHWD-870 was synthesized by Ningbo Wenda Pharma (Ninghai, Zhejiang, China).

1.3 Cell viability assay and combination treatment analysis

Cell viability was evaluated by the Cell Counting Kit-8 (CCK-8) (B34302, Bimake, USA). Cells with appropriate density per well were seeded in triplicate in 96-well plates and allowed to adhere. After cells were subjected to different treatments as indicated, the culture medium in each well of the plate was replaced with 100 μl fresh medium containing 10 μl CCK-8 solution. And the culture was returned to the incubator for 1-3 h at 37°C. Measure the absorbance at 450 nm using a microplate reader and calculate the cell viability according to the manufacturer’s instructions.

For evaluating the combination effect of BET inhibitors and RSL3 at indicated concentrations, CI values were calculated by CompuSyn software using Chou-Talalay method. CI values more than, equal to, or less than 1 indicate antagonistic, additive, or synergistic effects, respectively.

1.4 Colony formation assay

Three thousand A375 or SK-MEL-28 cells were seeded onto six-well plates per well for 2-3 d and then treated with the indicated reagents. The medium was replaced with fresh medium containing indicated drugs every 2-3 d. After 10-14 d, the colonies were fixed in 4% paraformaldehyde for 15 min and then stained with 0.5% crystal violet for 20 min. Next, remove the crystal violet mixture, wash the plates with tap water, and leave the colonies to dry in normal air. Images were obtained with a camera and colony numbers were counted with Image J software (National Institutes of Health, USA).

1.5 DNA constructs, lentiviral transduction and RNA interference

Single-guide RNAs (sgRNAs) targeting *GPX4*, *BRD4* and *AKR1C2* were cloned into the pLenti-CRISPR-V2 plasmid. The guide RNA sequences constituted a pool of two different sgRNA plasmids to target human *BRD4*. The following sgRNA sequences were used: sg*GPX4*, 5′-GGTGAAGCGCTACGGACCCA-3′; sg*BRD4*, 5′-CTCGTGAATGGGGTCAATGG-3′, 5′-CAAGATGCCGGACGAGCCTG-3′; sg*AKR1C2*, 5′-GGCTTCTATTGCCAATTTGA-3′, 5′-GATCCCATCGAGAAGAACCA-3′, 5′-ATTACCATGGTTCTTCTCGA-3′. Short hairpin RNA (shRNA) targeting *AKR1C2* was cloned into pLKO.1-EGFP-puro-shRNA plasmid and the shRNA sequence was: 5′-AGCTCTAGAGGCCGTCAAATT-3′. All constructs were verified by DNA sequencing. Moreover, shRNA vectors to deplete *BRD2/3/4* were purchased from Obio Technology (Shanghai, China). *STAT3* shRNA was purchased from GeneChem (Shanghai, China). BRD4 and AKR1C2 vector were purchased from Youze Biotechnology (Changsha, China). Briefly, to generate stable melanoma cell lines, HEK293T cells were transfected with lentivirus expression vector and lentivirus packaging plasmid (Addgene) mix for 48 h using TurboFect (R0531, Thermo Fisher Scientific, USA). The supernatant containing virus was collected, filtered, and added to culture melanoma cells for additional 48 h. Then the infected target cells were selected in the presence of 2 μg/mL puromycin (Thermo Fisher Scientific, USA) for 3 d. For transient transfection, knockdown of *BRD4* with siRNA was performed by transfection with si*BRD4* (GenePharma, China) using TurboFect according to the manufacturer’s protocol.

1.6 Lipid peroxidation assay

Lipid peroxidation production in melanoma cells was examined by C11-BODIPY

581/591 probes (D3861, Thermo Fisher Scientific, USA). For detection by flow cytometry, cells were seeded in six-well plates, treated with the indicated compounds, and then collected for staining with 2.5 μmol/L C11-BODIPY 581/591 dye at 37 °C for 30 min. Cells were washed with PBS twice to remove excess dye and resuspended with 500 μl of PBS. Finally, lipid peroxidation levels were tested by flow cytometry and analyzed by Flow Jo software (version 10.4, USA).

For lipid ROS detection by immunofluorescence, cells were inoculated onto the confocal dishes (BS-20-GJM, Biosharp, China), incubated with 2.5 μmol/L C11-BODIPY 581/591 at 37 °C for 30 min after indicated drug treatments, and then washed with PBS 2-3 times. The nuclei were stained with Hoechst staining solution for live cells (C1027, Beyotime Biotechnology, China) for 10 min at room temperature. The fluorescence images were visualized and pictured with a confocal microscope (Zeiss, Jena, Germany).

1.7 Transmission electron microscopy

A375 cells subjected to different treatments were fixed with 2.5% glutaraldehyde in Millonig's phosphate buffer (PH 7.3), and then incubated with 1% osmium tetroxide for 1 hour. After dehydration by increasing concentrations of acetone, the samples were embedded in 1:1 mix of acetone: resin for 12 h, and polymerized in 100% resin overnight at 37 °C and then at 60 °C for 12 h. Ultrathin sections of samples were cut using a Leica Ultracut microtome (Leica EM UC7, Germany) and stained with uranyl acetate and lead nitrate. Finally, the specimens were examined under a Hitachi HT-7700 electron microscope.

1.8 RNA extraction and Quantitative real-time PCR (qPCR)

Total RNA was isolated with Magzol reagent (R4801, Magen, China) and 1 μg of total RNA was used for cDNA synthesis using HiScript Q RT SuperMix kit (R223-01, Vazyme, China) according to the manufacturer's instruction. QPCR was conducted with SYBR Green qPCR Master Mix (B21703, Bimake, USA) in Applied Biosystems QuantStudio™ 3 Real-Time PCR System (Thermo Fisher Scientific, USA). Differences in mRNA expression were calculated by the ΔΔCt method using the housekeeping gene *ACTIN* as an internal reference control. All primer sequences used for qPCR were listed as follows:

BRD2-F: CGGCTTATGTTCTCCAACTGCTA;

BRD2-R: GGCAGTAGAGACTGGTAAAGGC;

BRD3-F: CCAACCATCACTGCAAACGTCAC;
BRD3-R: GGAGTGGTTGTGTCTGCTTTCC;
BRD4-F: CGCTATGTCACCTCCTGTTTGC;
BRD4-R: ACTCTGAGGACGAGAAGCCCTT;
AKR1C2-F: ATGCCTGTCCTGGGATTTGG;
AKR1C2-R: TCAATATGGTGGAACCCGGC;
ACTIN-F: CACCATTGGCAATGAGCGGTTC;
ACTIN-R: AGGTCTTTGCGGATGTCCACGT

1.9 Western blotting

Western blotting was performed according to the manufacturer’s instruction. Images were obtained by Odyssey® Fc Imaging System (LI-COR Biosciences, Lincoln, NE, USA) and quantitation of band intensity were performed by Image J software. The primary antibodies used in this study were: BRD2 (ab139690, Abcam, UK), BRD3 (ab264420, Abcam, UK), BRD4 (ab243862, Abcam, UK), AKR1C2 (13035S, Cell Signaling Technology, USA), Actin (sc-8432, Santa Cruz, USA), NRF2 (sc-365949, Santa Cruz, USA), AKR1C1 (ab192785, Abcam, UK), GPX4 (52455, Cell Signaling Technology, USA), STAT3 (9139, Cell Signaling Technology, USA), phospho-Stat3 (Tyr705) (9145, Cell Signaling Technology, USA), Flag (A8592, Sigma-Aldrich, USA).

1.10 Bioinformatics analysis

1.10.1 RNA-seq and data processing (related to Fig. 1f and Fig. S4a-c)

RNA-seq analysis was performed on A375 melanoma cell lines treated with the DMSO, BET inhibitor (JQ1), siControl or *BRD4* knockdown (si*BRD4*). Experiments were performed in biological duplicates. RNA-seq reads were adaptor trimmed and the data quality was assessed with the FastQC software (https://github.com/s-andrews/FastQC) before any data filtering criteria was applied. After obtaining the BAM files, reads were mapped onto the human reference genome (GRCh38.p12 assembly) by using HISAT2 software with default parameters. The mapped reads were assembled into transcripts or genes by using Stringtie software and the genome annotation file (hg38_ucsc.annotated.gtf). DESeq2 was used to analyze the difference between the two groups. Significant genes were identified by the criterion: |fold change| >= 1.5, adjust *P* value < 0.05.

1.10.2 Melanoma datasets collection (related to Fig. 1q-u, Fig. S3a and Fig. S8)

For melanoma datasets collection, mRNA expression, protein expression (PD-L1) and clinical data from skin cutaneous melanoma (SKCM) samples were downloaded from the TCGA data portal. Eleven GEO skin cutaneous melanoma cohorts were downloaded from Gene-Expression Omnibus (GEO; **Fig. S3a**) (https://www.ncbi.nlm.nih.gov/geo/). The richness of T cell receptor/B cell receptor (TCR/BCR) among TCGA-SKCM samples were available at https://gdc.cancer.gov/about-data/publications/panimmune. The Gide cohort (PRJEB23709: anti-PD-1 monotherapy and anti-PD-1/anti-CTLA-4 combined therapy) was downloaded from SRA database (https://www.ncbi.nlm.nih.gov/bioproject/). Processed gene expression profiles for melanoma single cell datasets were retrieved from TISCH (http://tisch.comp-genomics.org/) under accession numbers GSE72056, GSE115978, and GSE189889. The spatial data of melanoma obtained from BayesSpace package. The data was analyzed using R (version 3.6.0) and R bioconductor packages.

1.10.3 Estimate the ferroptosis status and analysis of drug response in ferroptosis status (related to Fig. 1a)

The ferroptosis score (FPS) was from the published algorithm model that is used for defining ferroptosis status by using R package (FPSOmics) (https://github.com/Yelab2020/FPSOmics). The FPS model was established based on the expression data for genes of core ferroptosis promoting components (pro-FRGs) including *ACSL4*, *ALOX5*, *NOX4*, *TF*, *ATG3*, *MAPK9*, *ATG5*, *BECN1*, *PRKAA1*, *SCP2*, *BACH1*, *HIF1A*, *MTDH*, *PI3KCA*, *SOCS1*, *TLR4*, *ATM*, *IFNG*, *ZEB1*, *FLT3*, *ATF3*, *MYB*, *SLC38A1*; and core negative ferroptosis components (anti-FRGs) of *SLC3A2*, *CBS*, *NQO1*, *PROM2*, *HSPB1*, *VDAC2*, *NF2*, *FH*, *BRD4*. We calculated enrichment score (ES) of pro-FRGs and anti-FRGs using single sample gene set enrichment analysis (ssGSEA) in the R package ‘GSVA’ (https://github.com/rcastelo/GSVA). The FPS to computationally dissect the ferroptosis status of the tissue samples, cancer cell line and single cell was defined by the differences of ssGSEA score between the ES of pro-FRGs minus anti-FRGs.

$$FPS = ssGSEA\_Score_{(pro\text{-}FRGs)} - ssGSEA\_Score_{(anti\text{-}FRGs)}$$

For analysis of drug response in ferroptosis status, the area under the dose-response curve (AUC) data, gene expression matrix for cancer cell lines and the drug repurposing information with drug-target were downloaded from the CTRP (https://portals.broadinstitute.org/ctrp.v2.1/). To assess the drug response associated

with ferroptosis status in cancer cell lines, we calculated the Spearman correlation between AUC data of drugs and ferroptosis score (|*r*| > 0.2; FDR < 0.05).

1.10.4 Single cell RNA-seq analysis of melanoma datasets (related to Fig. S8)

For single cell RNA-seq analysis of melanoma datasets, we merged all samples of corresponding cohort (GSE115978, GSE72056, and GSE189889) into a single Seurat object, and performed data pre-processing, normalization, integration, and clustering using the R package Seurat v3 and melanoma cells were then extracted for further analysis. We obtained and preprocessed the melanoma spatial sample using BayeSpace R package. Principal component analysis (PCA) was then performed on the top 2000 most highly variable genes. Then we modeled the top 15 PCs to dimension reduction. Single-cell ferroptosis scores were calculated using the FPS model algorithm described above. Uniform manifold approximation and projection (UMAP) embeddings of single-cell RNA-seq profiles show the annotated cell types, BRD4 and FPS expression. Spearman correlation was used to calculate the correlation coefficient between ferroptosis status and the expression of BRD4 in melanoma cells.

1.10.5 Calculating the immunotherapy outcome-related scores (related to Fig. S8h)

To calculate the immunotherapy outcome-related scores, T cell-inflamed gene expression profile (GEP) level in each sample was computed based on the gene signatures from Ayers et al by performing gene set variation analysis (GSVA). We obtained the cytolytic activity (CYT) level by calculating the geometric mean of the gene expression of two cytolytic markers (GZMA and PRF1). Other immune features, such as interferon-γ (IFN-γ) response, microsatellite instability (MSI) score, the infiltration of $CD8^+$ T cells, cytotoxic T lymphocytes (CTL), myeloid-derived suppressor cells (MDSCs) and the M2 subtype of tumor-associated macrophages (TAMs), T cell exclusion score in each melanoma sample was obtained based on the tumor immune dysfunction and exclusion (TIDE) computational framework (https://github.com/jingxinfu/TIDEpy).

1.11 Immunofluorescence

Melanoma cells were seeded on coverslips in 24 well plates and treated with indicated BET inhibitors for 48 h after attachment. The treated cells were fixed with 4% paraformaldehyde for 15 min and permeabilized with 0.2% Triton X-100/PBS for 10 min. Next, cells were blocked with 5% bovine serum albumin for 1 h at room temperature, followed by incubation with AKR1C2 antibody (13035S, Cell Signaling

Technology, USA) at 4 °C overnight. Cells were washed with PBS 3 times and treated with fluorescent secondary antibodies (Invitrogen, USA) at room temperature for 1 h and then sealed with a DAPI-containing fluoroshield mounting medium (ab104139, Abcam, UK). Finally, the fluorescence images were obtained with a confocal microscope (Zeiss, Jena, Germany) and Zeiss image software.

1.12 Chromatin immunoprecipitation (ChIP) sequencing

ChIP sequencing was conducted according to the standard protocols. Samples were immunoprecipitated with BRD4 (13440, Cell Signaling Technology, USA) antibody.

1.13 Immunohistochemistry

Xenograft tumor tissues were collected, fixed, embedded, and sectioned. For immunohistochemistry staining, the slides were stained with 4-hydroxynonenal (4-HNE) (ab46545, Abcam, UK) and GPX4 (52455, Cell Signaling Technology, USA) antibodies at 4 °C overnight. The next day, slides were incubated with a biotin-conjugated secondary antibody for 20 min and then with peroxidase-conjugated streptavidin for an additional 30 min. Next, diaminobenzidine was used to visualize the reaction, and slides were then counterstained with hematoxylin.

1.14 Flow cytometry analysis

For flow cytometry analysis of mouse samples, B16F10-xenograft tumor tissues were manually dissected and grinded. Single-cell suspensions were passed through 40 μm cell strainers (352340, BD Falcon, USA). After blocking cells with CD16/32 antibody (101320, Biolegend, USA) and identifying dead cells with Zombie Aqua^{TM} Fixable Viability Kit (423102, BioLegend, USA), cells were stained with antibody against the following: APC/Cyanine7-CD45 (103116, BioLegend, USA), APC-CD3 (100236, BioLegend, USA), PerCP/Cyanine5.5-CD4 (100434, BioLegend, USA), and PE/Cyanine7-CD8a (100722, BioLegend, USA) for 30 min. For further intracellular dyeing, cells were stimulated with Leukocyte Activation Cocktail (550583, BD Biosciences, USA) for 5 h, fixed and permeabilized by Foxp3/Transcription Factor Staining Buffer Set (00-5523-00, eBioscience, USA). Intracellular GZMB and IFN-γ was stained using PE/Dazzle^{TM} 594-Granzyme B (372216, BioLegend, USA) and FITC-IFN-γ (505805, BioLegend, USA) antibodies, respectively. Stained cells were detected by the DxP Athena^{TM} flow cytometry system (Cytek, USA) and the data were processed using Flow Jo software (version 10.4, USA).

1.15 Animal experiments

All experimental protocols and procedures involving mice were approved by the Ethical Review of the Animal Care and Use Committee of Central South University (No. 2022111244, Changsha, Hunan, China). Female BALB/c nude and C57BL/6 mice were obtained from Shanghai SLAC and were 4 - 6 weeks old. All mice were maintained under specific-pathogen-free conditions in the Animal Facility of the Central South University. To explore the combination effect of BET and GPX4 inhibition, GPX4 knockout or control A375 cells (2×10^6 cells per mouse) were suspended in 100 μl of PBS and inoculated subcutaneously into the right back flanks of immunodeficient nude mice. When tumor volumes approximately reached 100 mm^3, mice were randomly allocated into four subgroups: 1) injected with control A375 cells and treated with vehicle (corn oil, orally), 2) injected with GPX4 knockout A375 cells and treated with vehicle, 3) injected with control A375 cells and treated with NHWD-870 (in corn oil, 0.75 mg/kg, orally), and 4) injected with GPX4 knockout A375 cells and treated with NHWD-870. For BET inhibitors and immunotherapy combination therapy assay, 5×10^5 B16F10 cells per mouse were injected subcutaneously into the immunocompetent C57BL/6 mice. Tumor-bearing mice were randomly divided into four subgroups: 1) vehicle plus isotype IgG2α (BE0089, BioXcell, USA), 2) NHWD-870 plus isotype IgG2α, 3) vehicle plus anti-PD-1 antibody (BE0146, BioXcell, USA), and 4) NHWD-870 plus anti-PD-1 antibody. On day 6, 100 μg isotype IgG2α or 100 μg anti-PD-1 antibody was administered intraperitoneally to each mouse every 3 d. The administration route and dose of NHWD-870 or vehicle were same as above. The tumor volumes and body weight of all mice were monitored every 3 d. The tumor volumes were calculated as $0.5 \times \text{length} \times \text{width}^2$ based on calliper measurements.

1.16 Statistical analysis

Data were presented as mean ± SD and analyzed with GraphPad Prism software (version 8.0.1, San Diego, CA, USA). Two-tailed unpaired student's *t* test was used for comparison between two groups. ANOVA analysis with Tukey's multiple comparison test were employed for comparison among the different groups. Wilcoxon rank sum test was used to compare the differences of immunotherapy outcome-related scores between the high BRD4/AKR1C2 + low FPS group and the low BRD4/AKR1C2 + high FPS group. Fisher's exact test/chi-square test was performed to compare the proportion of patients with different responses to immunotherapy between the high BRD4/AKR1C2 +

low FPS group and the low BRD4/AKR1C2 + high FPS group. Kaplan-Meier comparative survival analyses for prognostic analysis were generated, and the log-rank test was used to determine the significance of the differences. All statistical analysis was two-side. *P* values of < 0.05 were considered statistically significant.

Supplementary figures

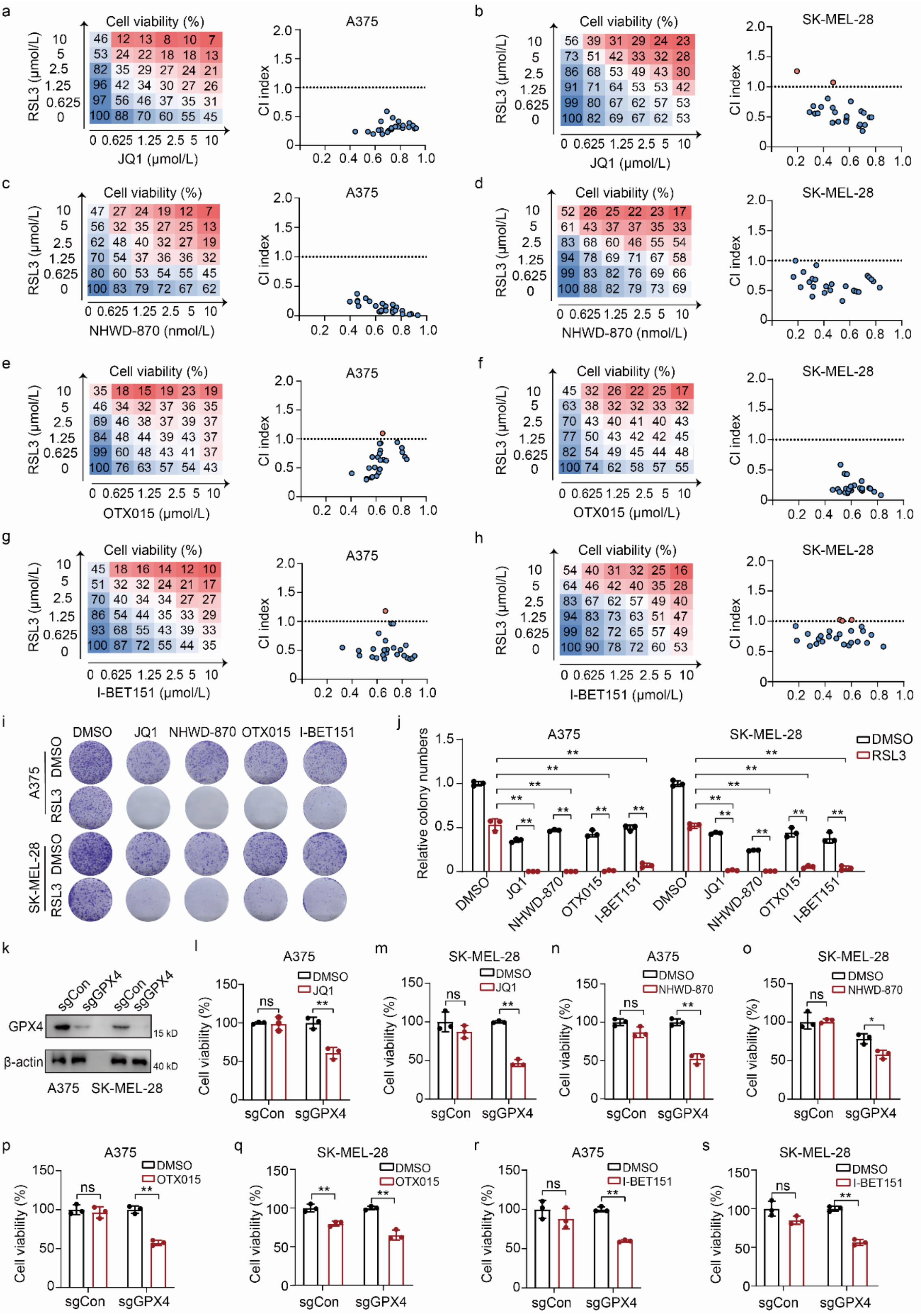

Fig. S1 BET inhibitors synergize with GPX4 inhibition in melanoma cells. **a-h** Relative cell viability and combination index (CI) of combination treatments were exhibited by the 6×6 screening experiments between four BET inhibitors JQ1 (**a-b**), NHWD-870 (**c-**

d), OTX015 (**e-f**) and I-BET151 (**g-h**) and ferroptosis inducer RSL3, respectively. A375 and SK-MEL-28 melanoma cells were pretreated with indicated doses of BET inhibitors for 48 h and then cotreated with RSL3 for 10 h for this 6×6 experiment. The *x*-axis of the CI plots indicates the percentage of cells affected. CI = 1 additive, CI < 1 synergistic, and CI > 1 antagonistic. **i-j** Representative images (**i**) and quantification (**j**) of colony formation assay in A375 and SK-MEL-28 melanoma cells treated with DMSO, BET inhibitors, RSL3 or combinations as indicated. JQ1, 0.1 μmol/L; NHWD-870, 1.5 nmol/L; OTX015, 0.1 μmol/L; I-BET151, 0.5 μmol/L; RSL3, 0.2 μmol/L. **k** Knockout efficiency of GPX4 with sgRNA was evaluated by western blotting analysis in A375 and SK-MEL-28 melanoma cells. **l-s** Relative cell viability of control (sgCon) and GPX4 knockout (sgGPX4) A375 or SK-MEL-28 cells treated with DMSO or 1 μmol/L JQ1 (**l-m**), 10 nmol/L NHWD-870 (**n-o**), 1 μmol/L OTX015 (**p-q**), 1 μmol/L I-BET151 (**r-s**) for 24 h. Quantification data are presented as mean ± SD ($n = 3$ biologically independent samples) and compared using two-way ANOVA in **j** and two-tailed t-test in **l-s**. BET bromodomain and extra-terminal domain, CI combination index, GPX4 glutathione peroxidase 4, ns not significant, * $P < 0.05$, ** $P < 0.01$.

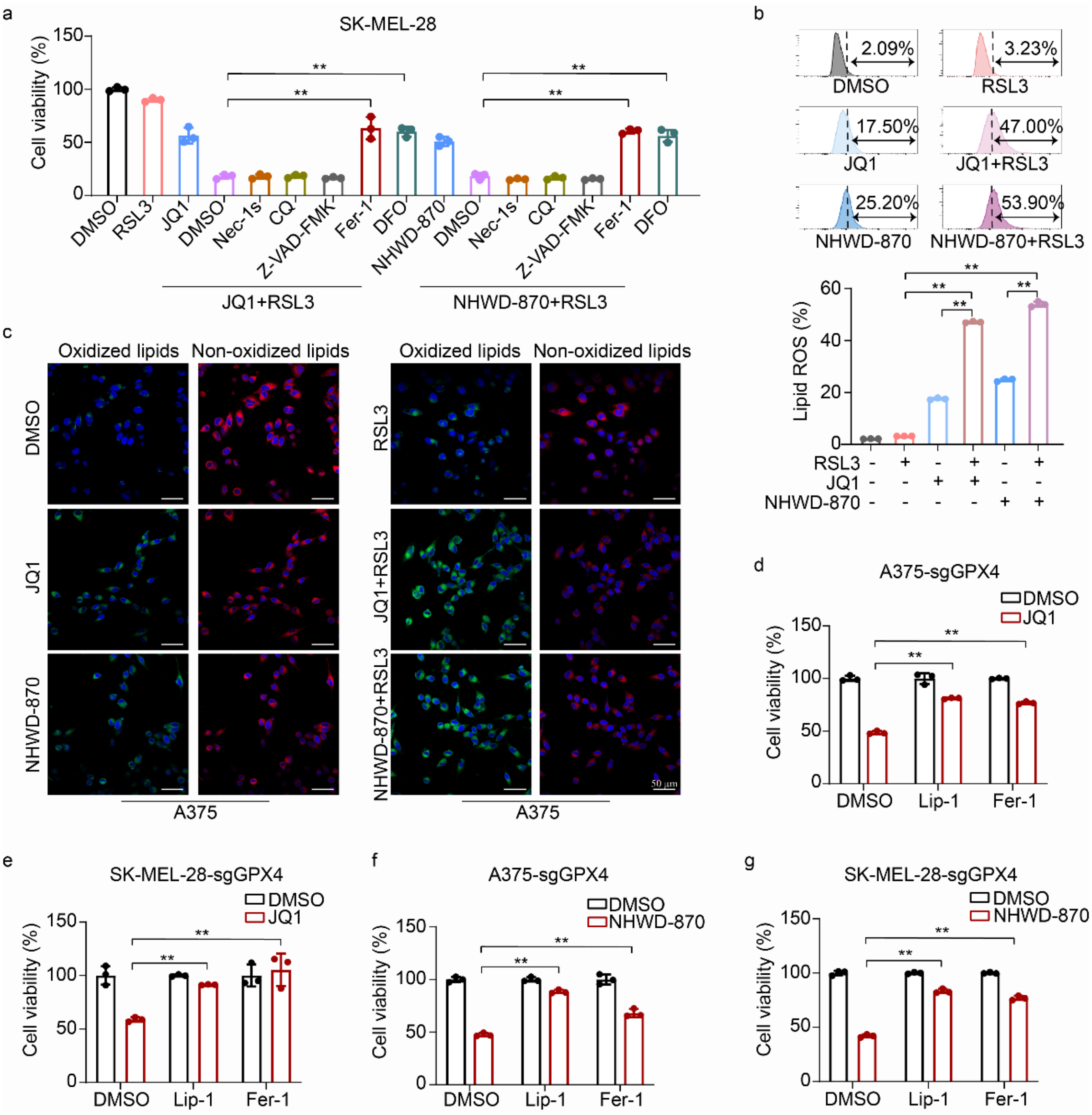

Fig. S2 Combination of BET inhibitors and GPX4 inhibition causes melanoma ferroptosis. **a** SK-MEL-28 melanoma cells were pretreated with JQ1 (1 μmol/L) or NHWD-870 (10 nmol/L) for 24 h, and then cotreated with RSL3 (2.5 μmol/L). DMSO, necrostatin-1s (Nec-1s, 10 μmol/L), chloroquine (CQ, 10 μmol/L), Z-VAD-FMK (10 μmol/L), ferrostatin-1 (Fer-1, 4 μmol/L), or deferoxamine (DFO, 100 μmol/L) were added in combination groups for 10 h, and cell viability was assessed. **b** Lipid peroxidation production in SK-MEL-28 cells were measured by flow cytometry using BODIPY-C11. Cells were treated with 1 μmol/L JQ1 or 10 nmol/L NHWD-870 for 24 h, alone or in combination with 2 μmol/L RSL3 for 6h as indicated. **c** Oxidized and non-oxidized lipids in A375 cells were shown by fluorescence images using the same BODIPY-C11 probes and treatments with **b**. Scale bar=50 μm. **d-g** Relative cell viability of GPX4 deficient A375 and SK-MEL-28 cells subjected to 1μmol/L JQ1(**d-e**)

or 10 nmol/L NHWD-870 (**f-g**) treatments with or without liproxstatin-1 (Lip-1, 10 μmol/L) or 4 μmol/L Fer-1. Quantification data are presented as mean ± SD (*n* = 3 biologically independent samples) and compared with one-way ANOVA in **a-b**, **d-g**. BET bromodomain and extra-terminal domain, CQ chloroquine, DFO deferoxamine, Fer-1 ferrostatin-1, GPX4 glutathione peroxidase 4, Lip-1 liproxstatin-1, Nec-1s necrostatin-1s, * $P < 0.05$, ** $P < 0.01$.

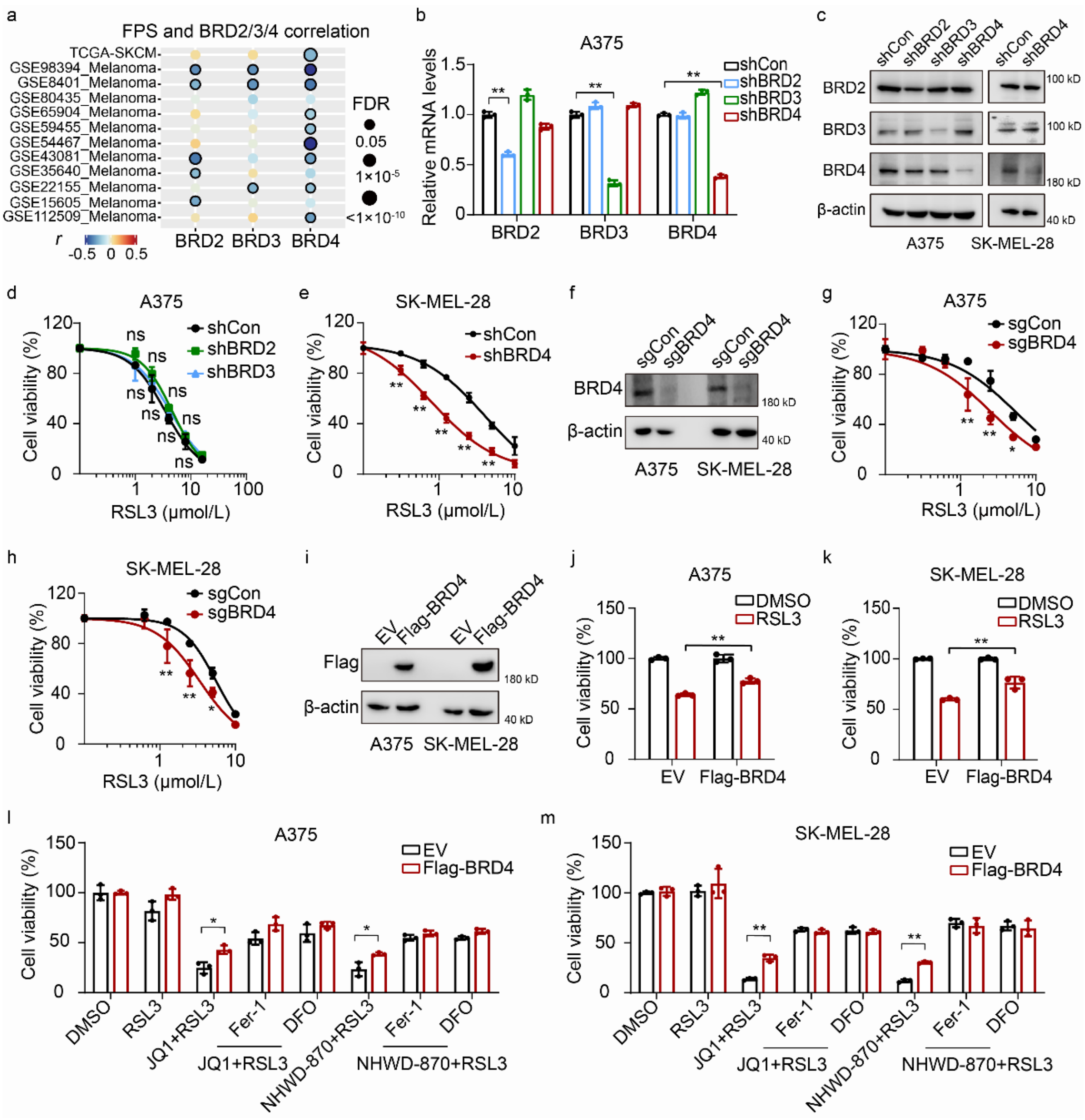

Fig. S3 BET inhibitors sensitize melanoma cells to RSL3-induced ferroptosis by targeting BRD4. **a** Heatmaps show the Spearman's rank correlation between ferroptosis score (FPS) and mRNA expression of BRD2/3/4 among 12 melanoma datasets. The color intensity indicates Spearman correlation coefficient (*r*); the dot size indicates FDR for the Spearman correlation. The black circle border indicates FDR < 0.05. **b-c** The knockdown efficiency of BRD2, BRD3 and BRD4 with shRNAs in melanoma cells was assessed by real-time PCR (**b**) and western blotting analysis (**c**). **d** Dose-response curves of RSL3-induced death of control (shCon), BRD2 knockdown (shBRD2) and BRD3 knockdown (shBRD3) A375 cells. **e** Dose-response curves of RSL3-induced death of shCon and BRD4 knockdown (shBRD4) SK-MEL-28 cells. **f** BRD4 protein levels were quantified by western blotting in control (sgCon) and BRD4-deficent (sgBRD4) melanoma cells. **g-h** Dose-response curves of RSL3-induced death of sgCon and

sgBRD4 A375 (**g**) or SK-MEL-28 (**h**) cells. **i** BRD4 protein levels were examined by western blotting in control (Flag vector, EV) and BRD4 overexpression (Flag-BRD4) A375 or SK-MEL-28 cells. **j-k** Relative viability of control (EV) and BRD4 overexpression (Flag-BRD4) A375 (**j**) or SK-MEL-28 cells (**k**) treated with DMSO or 2.5 μmol/L RSL3 for 10h. **l-m** Relative viability of control (EV) and BRD4 overexpression (Flag-BRD4) A375 (**l**) or SK-MEL-28 cells (**m**) treated with 2.5 μmol/L RSL3 alone or plus BET inhibitor in the absence or presence of Fer-1 (4 μmol/L) or DFO (100 μmol/L). JQ1, 1 μmol/L; NHWD-870, 10 nmol/L. Quantification data are presented as mean ± SD ($n = 3$ biologically independent samples) and compared with one-way ANOVA in **b**, and two-way ANOVA in **d-e**, **g-h**, two-tailed t-test in **j-m**. BET bromodomain and extra-terminal domain, BRD bromodomain-containing protein, DFO deferoxamine, Fer-1 ferrostatin-1, FPS ferroptosis score, ns not significant, * $P < 0.05$, ** $P < 0.01$.

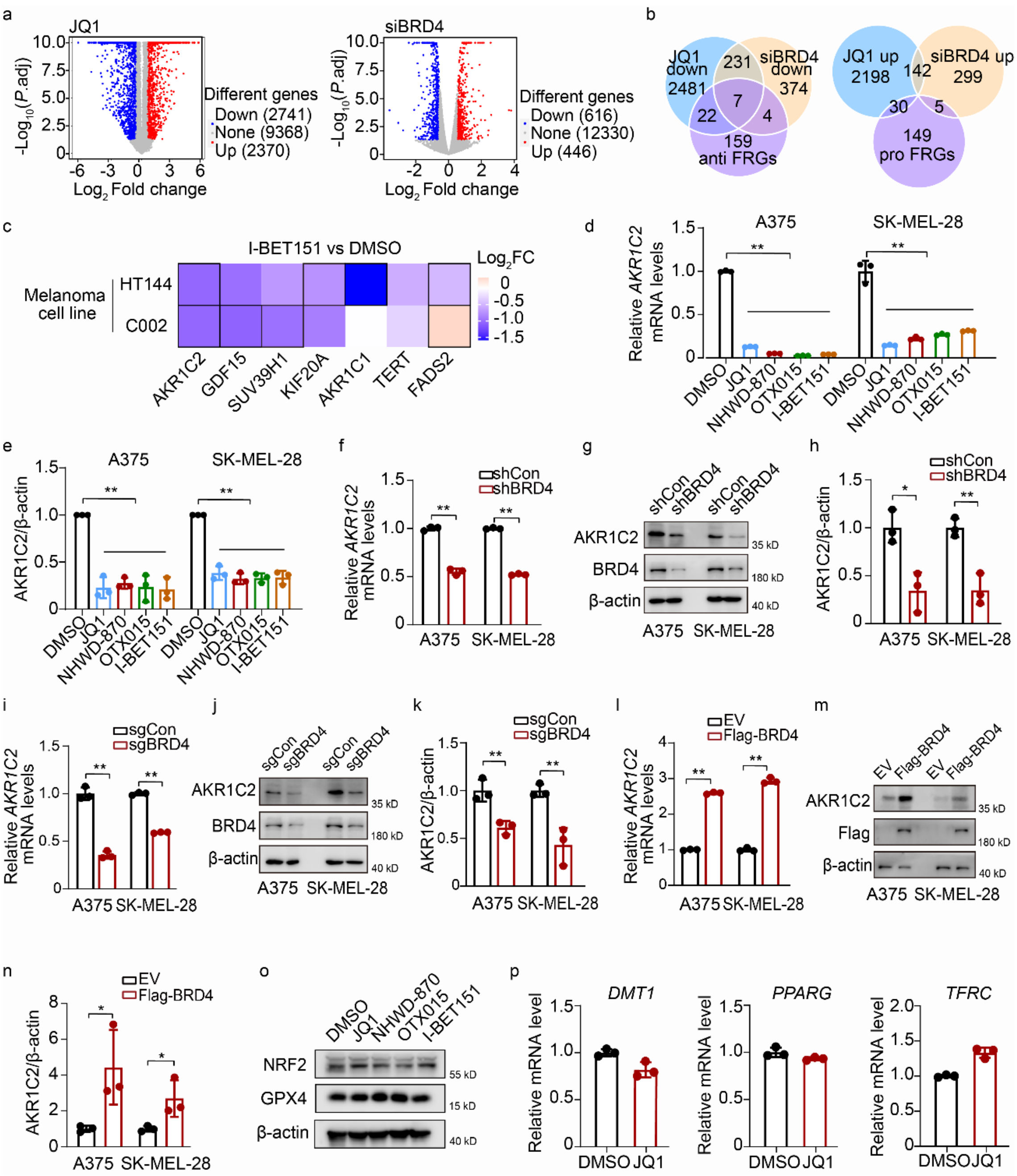

Fig. S4 BET inhibitors targeted BRD4 regulate AKR1C2 expression by directly targeting its promoter. **a** Volcano plot shows the changes in gene expression from RNA-seq data after JQ1 treated or BRD4 knockdown (siBRD4) versus control. **b** Venn diagram displaying numbers of genes significantly downregulated (left panel) or upregulated (right panel) after JQ1 or siBRD4 treated and the overlap with the ferroptosis suppressor genes (anti-FRGs, left panel) or ferroptosis driver genes (pro-FRGs, right panel). **c** Heatmap displayed the changes in 7 overlap genes expression in I-BET151-treated over DMSO-treated melanoma cell lines (GSE167241). The black square border represents FDR < 0.05. **d** *AKR1C2* mRNA levels in A375 and SK-MEL-28 cells after indicated BET inhibitor treatment for 48 h. JQ1, 2 μmol/L; NHWD-870,

10 nmol/L; OTX015, 2 μmol/L; I-BET151, 2 μmol/L. **e** Quantitative analysis of AKR1C2 expression after the same BET inhibitor treatment as **d** by western blotting. **f** *AKR1C2* mRNA levels in control (shCon) and BRD4 knockdown (shBRD4) melanoma cells. **g-h** Western blotting and quantitative analysis of AKR1C2 expression in shCon and shBRD4 melanoma cells. **i** *AKR1C2* mRNA levels in control (sgCon) and BRD4 knockout (sgBRD4) melanoma cells. **j-k** Western blotting and quantitative analysis of AKR1C2 expression in sgCon and sgBRD4 melanoma cells. **l** *AKR1C2* mRNA levels in control (EV) and BRD4 overexpression (BRD4) melanoma cells. **m-n** Western blotting and quantitative analysis of AKR1C2 expression in EV and BRD4 overexpression melanoma cells. **o** The effects of BET inhibitors on the expression of NRF2 and GPX4 in A375 cells treated for 48 h were analyzed by western blotting. JQ1, 2 μmol/L; NHWD-870, 10 nmol/L; OTX015, 2 μmol/L; I-BET151, 2 μmol/L. **p** RNA sequencing showing the mRNA expression of NRF2 target genes *DMT1*, *PPARG* and *TFRC* in A375 cells treated with DMSO or JQ1. Quantification data are presented as mean ± SD ($n = 3$ biologically independent samples) and compared with one-way ANOVA in **d-e** and two-tailed t-test in **f, h-i, k-l, n**. AKR1C2 aldo-keto reductase 1C2, BET bromodomain and extra-terminal domain, BRD4 bromodomain-containing protein 4, GPX4 glutathione peroxidase 4, anti-FRG ferroptosis suppressor gene, pro-FRG ferroptosis driver gene, * $P < 0.05$, ** $P < 0.01$.

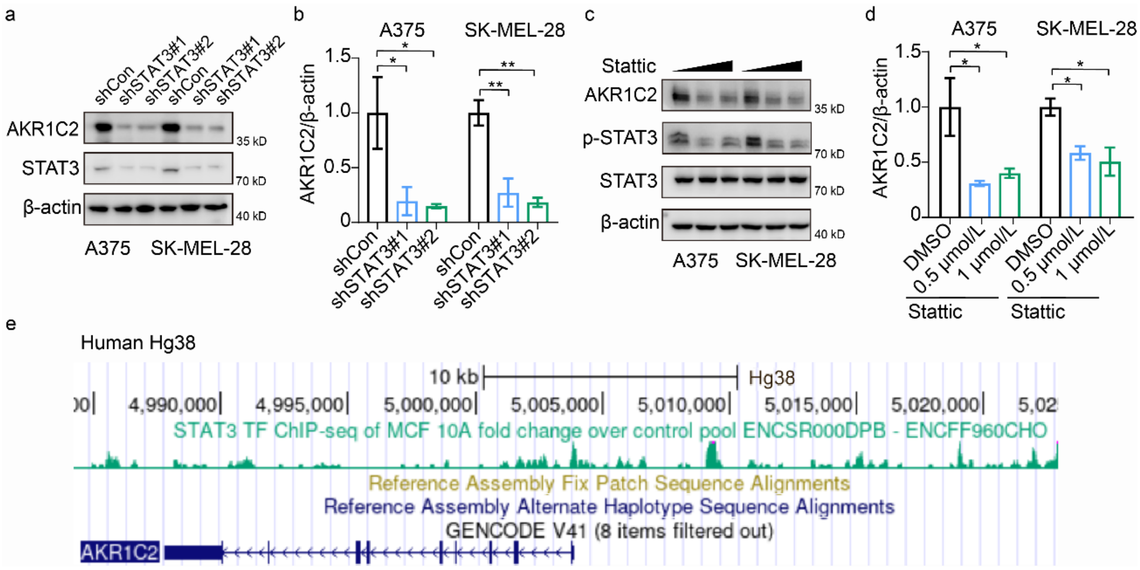

Fig. S5 BET inhibitors targeted BRD4 regulates AKR1C2 expression by indirectly targeting the IL6/STAT3 axis. **a-b** Western blotting and quantitative analysis of AKR1C2 expression in control (shCon) and *STAT3* knockdown (shSTAT3) melanoma cells. **c-d** Western blotting and quantitative analysis of AKR1C2 expression in DMSO- and stattic-treated melanoma cells. **e** ChIP-seq analysis of STAT3 binding peak in the *AKR1C2* promoter in MCF 10A cells. Quantification data are presented as mean ± SD ($n = 3$ biologically independent samples) and compared with one-way ANOVA in **b, d**. AKR1C2 aldo-keto reductase 1C2, STAT3 signal transducer and activator of transcription 3, * $P < 0.05$, ** $P < 0.01$.

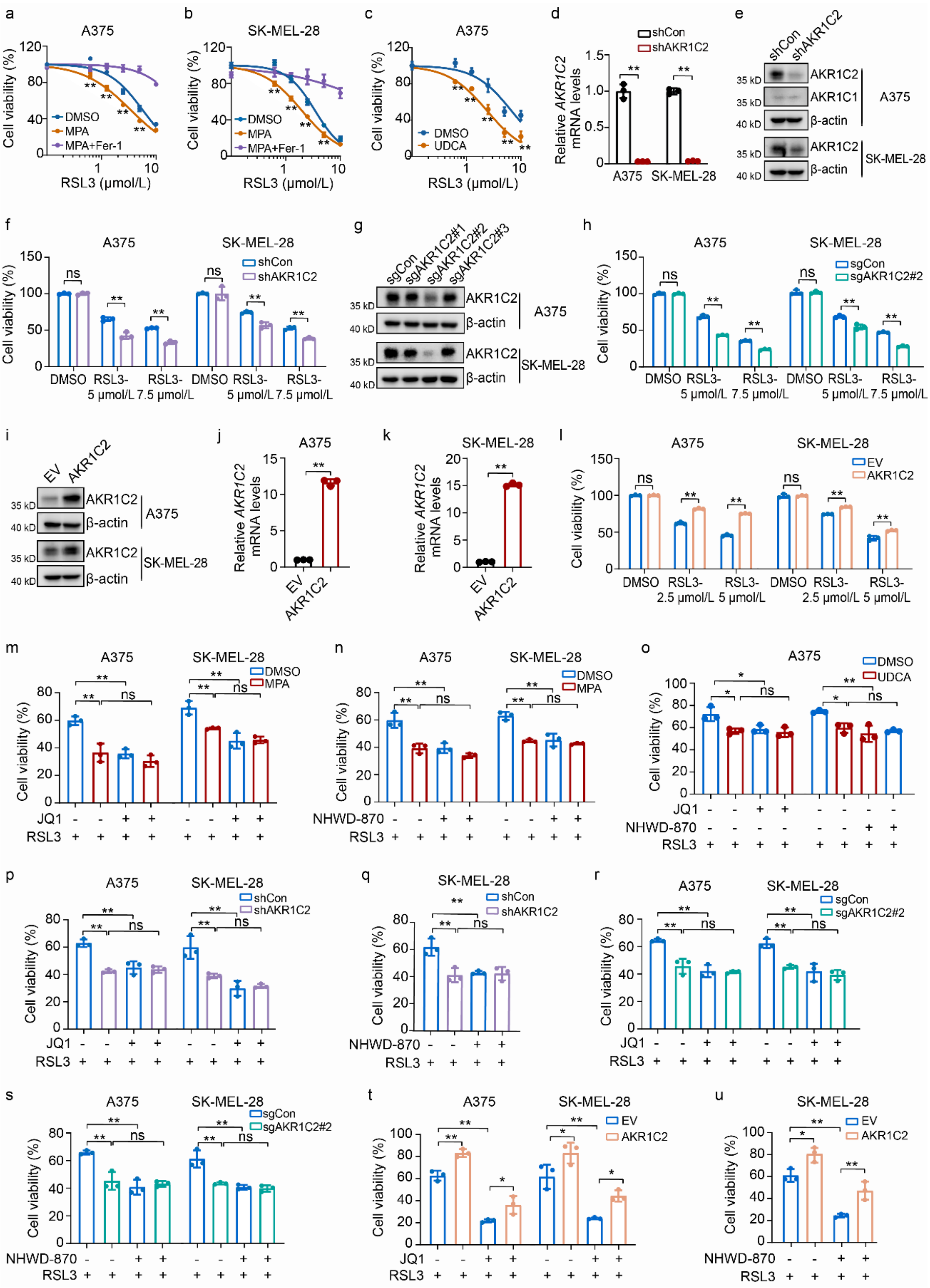

Fig. S6 BET inhibitors regulate melanoma susceptibility to RSL3-induced ferroptosis through AKR1C2. **a-b** Dose response of RSL3-triggered death of A375 (**a**) and SK-MEL-28 cells (**b**) in the presence of DMSO, AKR1C2 inhibitor (medroxyprogesterone acetate, MPA), or MPA plus Fer-1 for 10 h. MPA, 15 μmol/L; Fer-1, 4 μmol/L. **c** Dose response of RSL3-triggered death of A375 cells in the presence of DMSO or AKR1C2

inhibitor (ursodeoxycholate, UDCA) for 10 h. UDCA, 200 μmol/L. (**d-e**) Knockdown efficiency of silencing *AKR1C2* by shRNA was assessed by real-time PCR (**d**) and western blotting analysis (**e**). **f** Relative viability of control (shCon) and *AKR1C2* knockdown (shAKR1C2) A375 and SK-MEL-28 cells treated with different doses of RSL3 for 10h. **g** AKR1C2 protein levels in control (sgCon) and *AKR1C2* knockout (sgAKR1C2) melanoma cells. **h** Relative viability of sgCon and sgAKR1C2 A375 and SK-MEL-28 cells treated with different doses of RSL3 for 10 h. **i** AKR1C2 protein levels in control (EV) and AKR1C2 overexpression (AKR1C2) melanoma cells. **j-k** *AKR1C2* mRNA levels in control (EV) and AKR1C2 overexpression (AKR1C2) A375 (**j**) and SK-MEL-28 (**k**) cells. **l** Relative viability of EV and AKR1C2 overexpression A375 and SK-MEL-28 cells treated with different doses of RSL3 for 10 h. **m-n** Relative viability of 5 μmol/L RSL3-treated A375 and SK-MEL-28 cells in the presence of DMSO, MPA, JQ1, JQ1 plus MPA (**m**) or in DMSO, MPA, NHWD-870, NHWD-870 plus MPA (**n**). MPA, 15 μmol/L; JQ1, 2 μmol/L; NHWD-870, 10 nmol/L. **o** Relative viability of 2.5 μmol/L RSL3-treated A375 cells in the presence of DMSO, UDCA, JQ1, JQ1 plus UDCA, NHWD-870, or NHWD-870 plus UDCA. UDCA, 200 μmol/L; JQ1, 2 μmol/L; NHWD-870, 10 nmol/L. **p-q** Relative viability of 5 μmol/L RSL3-treated shCon and shAKR1C2 A375 or SK-MEL-28 cells in the presence of DMSO, 2 μmol/L JQ1 (**p**) or DMSO, 10 nmol/L NHWD-870 (**q**). **r-s** Relative viability of 5 μmol/L RSL3-treated sgCon and sgAKR1C2 A375 and SK-MEL-28 cells in the presence of DMSO, 2 μmol/L JQ1 (**r**) or DMSO, 10 nmol/L NHWD-870 (**s**). **t-u** Relative viability of 5 μmol/L RSL3-treated EV and AKR1C2 overexpression A375 or SK-MEL-28 cells in the presence of DMSO, 2 μmol/L JQ1 (**t**) or DMSO, 10 nmol/L NHWD-870 (**u**). Quantification data are presented as mean ± SD ($n = 3$ biologically independent samples) and compared with two-way ANOVA in **a-c**, **m-u**, and two-tailed t test in **d**, **f**, **h**, **j-l**. Fer-1 ferrostatin-1, MPA medroxyprogesterone acetate, UDCA ursodeoxycholate, ns not significant, * $P < 0.05$, ** $P < 0.01$.

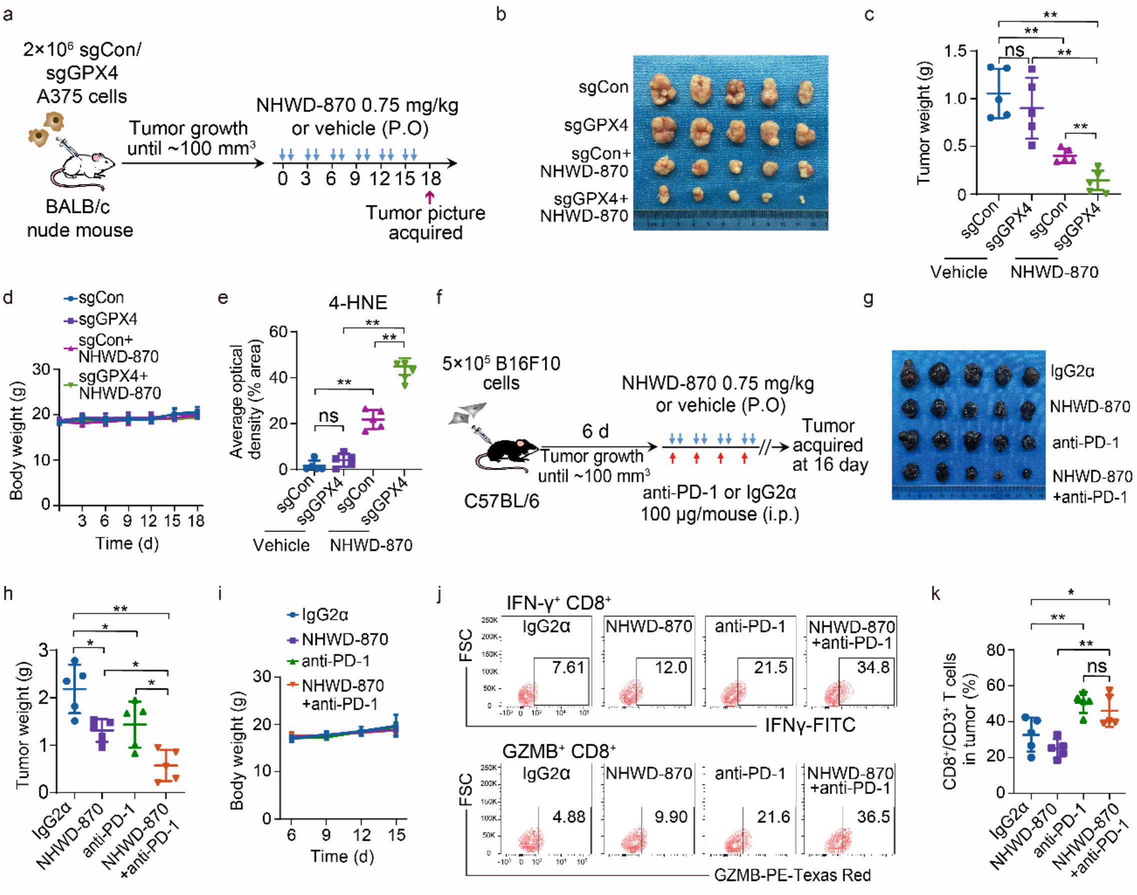

Fig. S7 BET inhibitors potentiate melanoma ferroptosis induced by GPX4 inhibition and immunotherapy in vivo. **a** Schematic outline of the xenograft experiments: control or *GPX4* knockout A375 cells were subcutaneously injected into female nude mice. When tumors reached 100 mm^3, the tumor-bearing mice were orally treated with vehicle or NHWD-870 (0.75 mg/kg) at indicated d. **b** Images of control and *GPX4* knockout A375 xenograft tumors from mice following the last measurement of tumor volumes. **c-d** Average tumor weight (**c**) and body weight of mice (**d**) in the indicated groups. **e** Quantification of 4-hydroxynonenal (4-HNE) in immunohistochemistry staining by Image J. **f** Schedule for administration of NHWD-870 (0.75 mg/kg) and anti-PD-1 (100 μg/per mouse) in B16F10 tumor-bearing C57BL/6 mice. **g** Images of B16F10 xenograft tumors from mice following the last measurement of tumor volumes in the BET inhibition and immunotherapy combination. **h-i** Average tumor weight (**h**) and body weight of mice (**i**) in the indicated groups. **j** The percentage of cells expressing IFN-γ and GZMB in tumor-infiltrating CD8$^+$ T cells by flow cytometry analysis. **k** The percentage of CD8$^+$ T cells in tumor-infiltrating CD3$^+$ T cells by flow cytometry analysis. Quantification data are presented as mean ± SD and compared with one-way ANOVA in **c**, **e**, **h**, **k**. GPX4 glutathione peroxidase 4, GZMB granzyme B, 4-HNE 4-

hydroxynonenal, IFN-γ interferon-γ, ns not significant, * $P < 0.05$, ** $P < 0.01$.

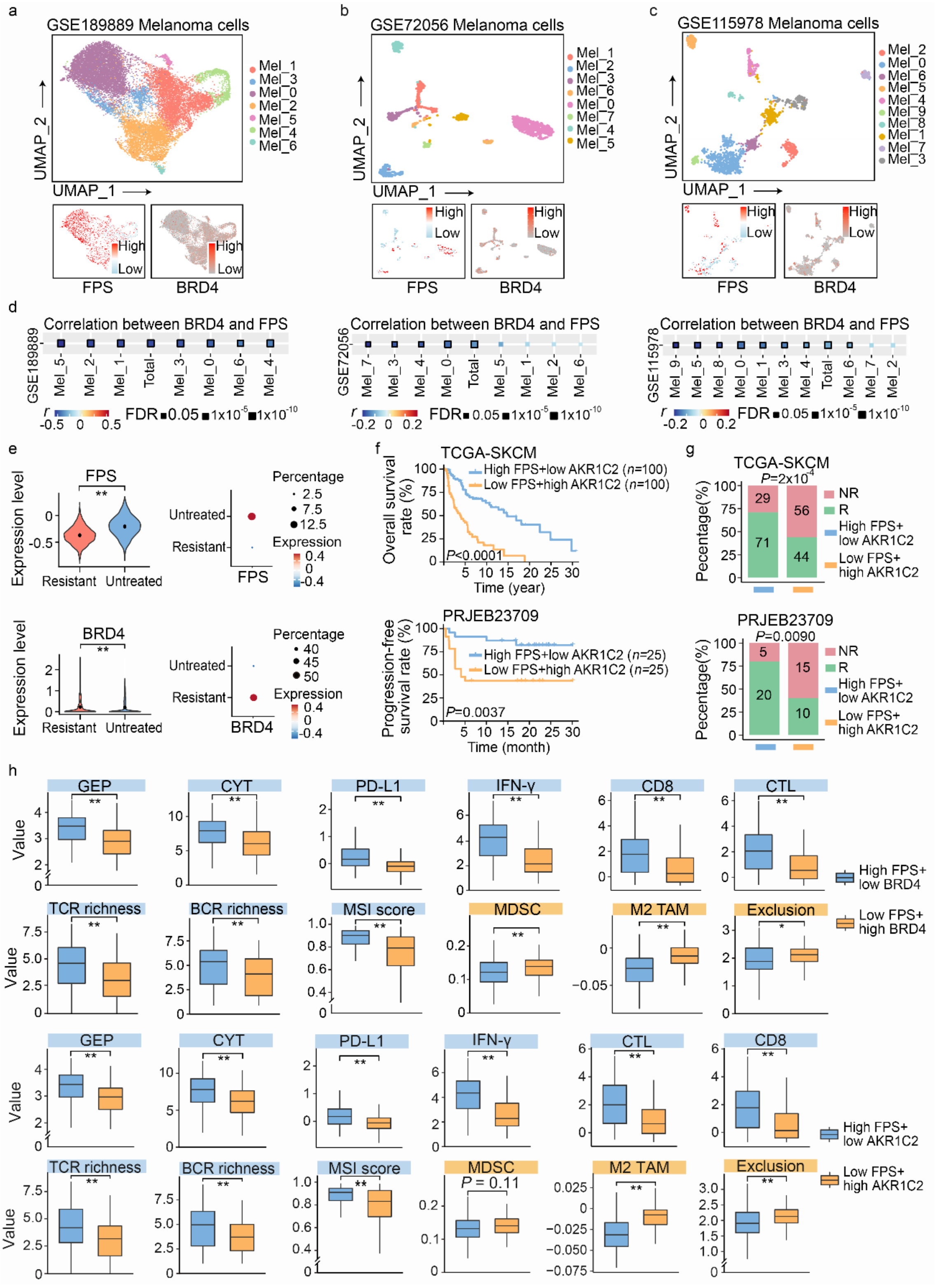

Fig. S8 BRD4/AKR1C2 is associated with reduced ferroptosis level and poor efficacy of immunotherapy from multi-omics characterization. **a-c** Uniform manifold

approximation and projection (UMAP) plot showing clusters of tumor cell subsets among three melanoma datasets (GSE189889; GSE72056; GSE115978). The bottom panel shows the expression levels of BRD4 and ferroptosis score (FPS) in melanoma subtypes. **d** The Spearman correlation between FPS and mRNA expression of BRD4 among 3 single cell datasets of melanoma cells. The color intensity indicates Spearman correlation coefficient (*r*); the dot size indicates FDR for the Spearman correlation. The black circle border indicates FDR < 0.05. **e** VlnPlot (left panel) and DotPlot (right panel) show the differences of BRD4 and FPS between immunotherapy resistant malignant cells and untreated malignant cells in GSE115978. **f** Kaplan-Meier curves compare overall survival (TCGA-SKCM) and progression-free survival of immune checkpoint inhibitors (ICIs) cohorts (PRJEB23709) between the high-FPS + low AKR1C2 (blue) and low-FPS + high AKR1C2 (yellow) groups. **g** The proportion of patients with different responses to immunotherapy in the TCGA-SKCM cohort with TIDE-predicted ICB response and the melanoma ICIs cohort (PRJEB23709). R: Response, including responder, complete response and partial response; NR: non-Response, including non-responder, stable disease and progressive disease. **h** The differences of immunotherapy outcome-related scores between the high FPS + low BRD4 group (blue) and the low FPS + high BRD4 group (yellow) (top panel) or between the high FPS + low AKR1C2 group (blue) and the low FPS + high AKR1C2 group (yellow) (down panel). Statistical significance was assessed using the log-rank test (**f**) and Fisher's exact test/chi-square test (**g**). Wilcoxon rank sum test was used in **h**. AKR1C2 aldo-keto reductase 1C2, BRD4 bromodomain-containing protein 4, CTL cytotoxic T lymphocyte, CYT cytolytic activity, FPS ferroptosis score, GEP T cell-inflamed gene expression profile, GPX4 glutathione peroxidase 4, ICIs immune checkpoint inhibitors, IFN-γ interferon-γ, MDSC myeloid-derived suppressor cell, MSI microsatellite instability, NR non-Response, R response, SKCM skin cutaneous melanoma, TAM tumor-associated macrophage, TCGA The Cancer Genome Atlas, TIDE Tumor Immune Dysfunction and Exclusion, UMAP uniform manifold approximation and projection, * $P < 0.05$, ** $P < 0.01$.

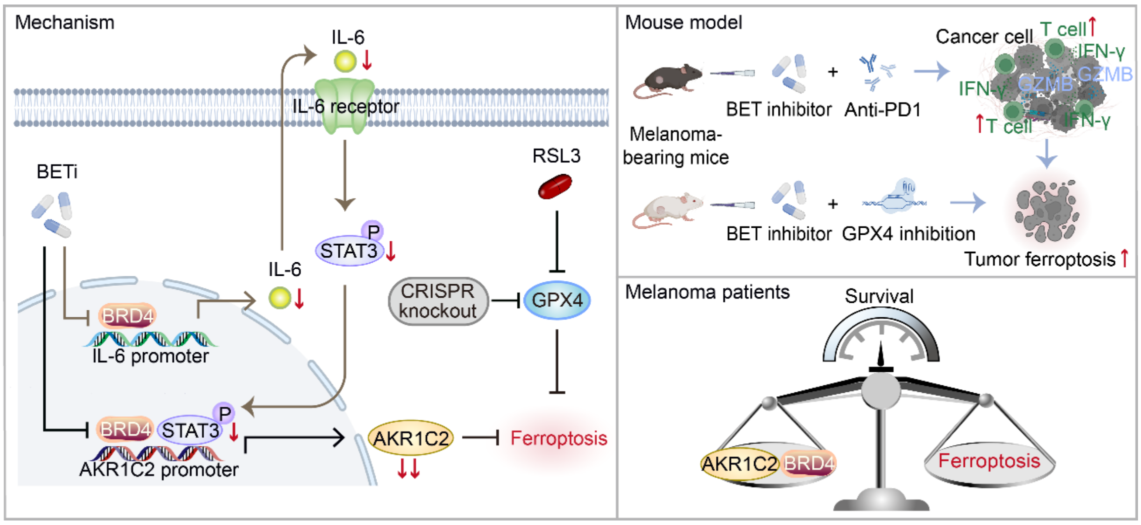

Fig. S9 Schematic depicting BET inhibitor-mediated sensitization to ferroptosis induced by GPX4 inhibition in melanoma cells. BET inhibitors suppress the ferroptosis suppressor AKR1C2 expression directly by BRD4 or indirectly by BRD4/IL6/STAT3 axis. In vivo, BET inhibitors synergize with GPX4 inhibition as well as ferroptosis-involved immunotherapy to suppress melanoma growth.
